# Case Report: Exploring the underlying mechanisms of psoriasis comorbid with sarcoidosis: insights from a case of coexisting immune-related disorders

**DOI:** 10.3389/fimmu.2025.1685695

**Published:** 2025-11-21

**Authors:** Yingbao Zhen, Xinlin Liang, Dawei Huang, Yangfeng Ding, Yuling Shi, Qian Yu

**Affiliations:** 1 Department of Dermatology, Shanghai Skin Disease Hospital, School of Medicine, Tongji University, Shanghai, China; 2 Institute of Psoriasis, School of Medicine, Tongji University, Shanghai, China

**Keywords:** sarcoidosis, psoriasis, coexistence, pathogenesis, immunity

## Abstract

Psoriasis, a chronic inflammatory disease characterized by hyperactivation of the Th1/Th17 axis, is increasingly recognized for its comorbid mechanisms with other immune-related disorders. This article reports a rare case. The case involves a 62-year-old woman with a 30-year history of chronic plaque psoriasis who developed sudden annular nodules on both lower limbs. Histopathology revealed non-caseating epithelioid granulomas, and PET-CT confirmed multi-system involvement, leading to a diagnosis of sarcoidosis. After three months of oral MTX therapy, the sarcoidosis skin lesions and psoriatic lesions showed significant improvement. The rare instance of coexistence suggests that chronic Th-cell immune activation may disrupt immune homeostasis through distinct pathways. This study presents clinical insights for an understanding of the mechanisms underlying immune-related comorbidities. It emphasizes the importance of implementing dynamic immune monitoring in the management of chronic inflammatory skin diseases

## Introduction

Psoriasis, a chronic relapsing inflammatory disease characterized by hyperactivation of the IL-23/Th17 axis, exhibits systemic effects extending beyond the cutaneous domain. This condition demonstrates associations with multi-system disorders (1). The pathogenic mechanisms underlying comorbidities in psoriasis patients remain incompletely elucidated, with shared immune pathways representing a potential contributing factor.

Sarcoidosis is a chronic, multisystem disorder of unknown etiology, characterized by the formation of non-necrotizing epithelioid cell granulomas. Studies indicate that sarcoidosis manifests as a Th1/Th17-mediated multisystem disorder, with cutaneous involvement occurring in approximately 30% of patients (2). Although the initial documentation of sarcoidosis and psoriasis coexistence in a single patient dates back to Winkelmann and Farmer’s report in the 1960s (3), the precise relationship between these disorders remains elusive. Consequently, dermatologists should maintain heightened vigilance to facilitate early diagnosis of both conditions and investigate their underlying pathogenic mechanisms. Studies have shown that sarcoidosis is also a Th1/Th17-mediated multisystem disorder—paralleling the immunopathology of psoriasis—and the role of Th1/Th17-driven inflammation in sarcoid granuloma formation is well-established (4–6). Multiple studies propose that psoriasis and sarcoidosis may share a TNF-α-mediated pathogenic mechanism. This potential commonality could explain the efficacy of TNF-α inhibitors in both diseases.

We report a case of psoriasis presenting with sarcoidosis, detailing the diagnosis and treatment process, aiming to provide a shared underlying pathogenic mechanism for understanding the mechanism behind this phenomenon.

## Case

A 62-year-old woman was admitted to our department with a two-week history of asymptomatic annular cutaneous nodules on both lower limbs. She presented with a 30-year history of plaque psoriasis and an 8-year history of hypertension. Her current medication included oral irbesartan 300 mg daily. A family history revealed psoriasis in her maternal grandmother.

**Figure 1:**
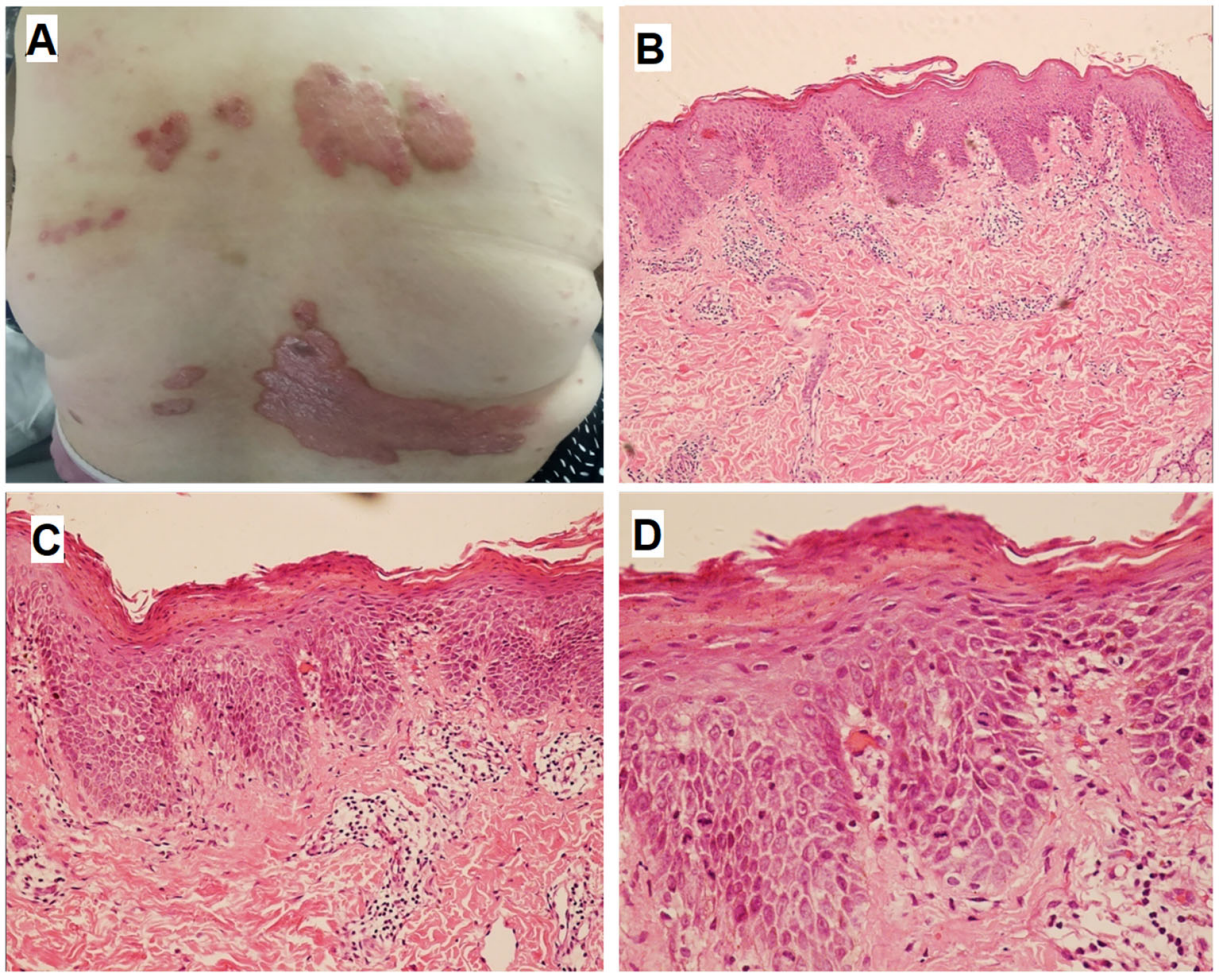
Skin lesions and histopathologic finding of psoriasis. Multiple well-demarcated erythematous plaques with overlying silvery scales were observed in the lumbosacral region **(A)**. HE staining revealed epidermal acanthosis, elongation of rete ridges, thinning of the suprapapillary epidermal plates, loss of the granular layer, and compact hyperkeratosis **(B)**, ×100; **(C)** , ×200; **(D)**, ×400).

**Figure 2:**
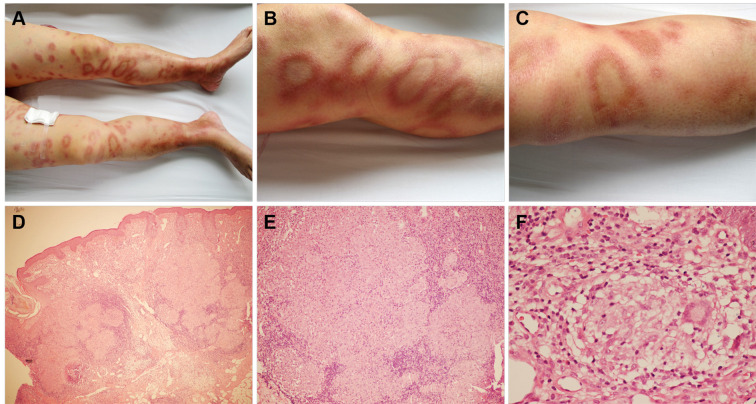
Skin lesions and histopathologic finding of sarcoidosis. Multiple erythematous nodules with partial annular configurations were observed on both lower extremities **(A-C)**. Histological examination demonstrated focal epithelioid granulomas accompanied by multinucleated giant cells in the subcutaneous tissue, with sparse lymphocytic infiltration and absence of caseous necrosis **(D)** ×100; **(E)** ×200; **(F)** ×400).

Physical examination demonstrated well-demarcated erythematous plaques covered by silvery scales in the lumbosacral region (**Figure 1A**). Multiple erythematous nodules, partially arranged in annular configurations, were observed on both lower extremities (**Figure 2A-C**). Palpation revealed a nontender, enlarged right cervical lymph node with a smooth surface. No other significant dermatological or systemic abnormalities were identified.

Laboratory investigations revealed an elevated ESR of 65 mm/h (reference range: 0 to 20 mm/h) and a CRP level of 102 mg/L (reference range: 0 to 10 mg/L). All other laboratory results, including serum CEA, CA 15-3, CA 19-9, neuron-specific enolase (NSE), serum complement analysis, RF, anti-CCP antibody, ANA, ANCA, immunoglobulin levels, angiotensin-converting enzyme (ACE), serological testing for hepatitis A, B, and C viruses, and HIV testing, were within normal limits. A whole blood interferon-gamma release assay (IGRA) for *Mycobacterium tuberculosis* antigens was negative. Counts of erythrocytes: 4.6×10¹²/L; Counts of leukocytes: 8.2×10^9^/L; Counts of platelets: 248×10^9^/L; Hemoglobin: 140 g/L; Calcium: 2.4 mmol/L; Phosphorus: 1.23 mmol/L. No other clinically significant abnormalities were identified.

**Figure 3:**
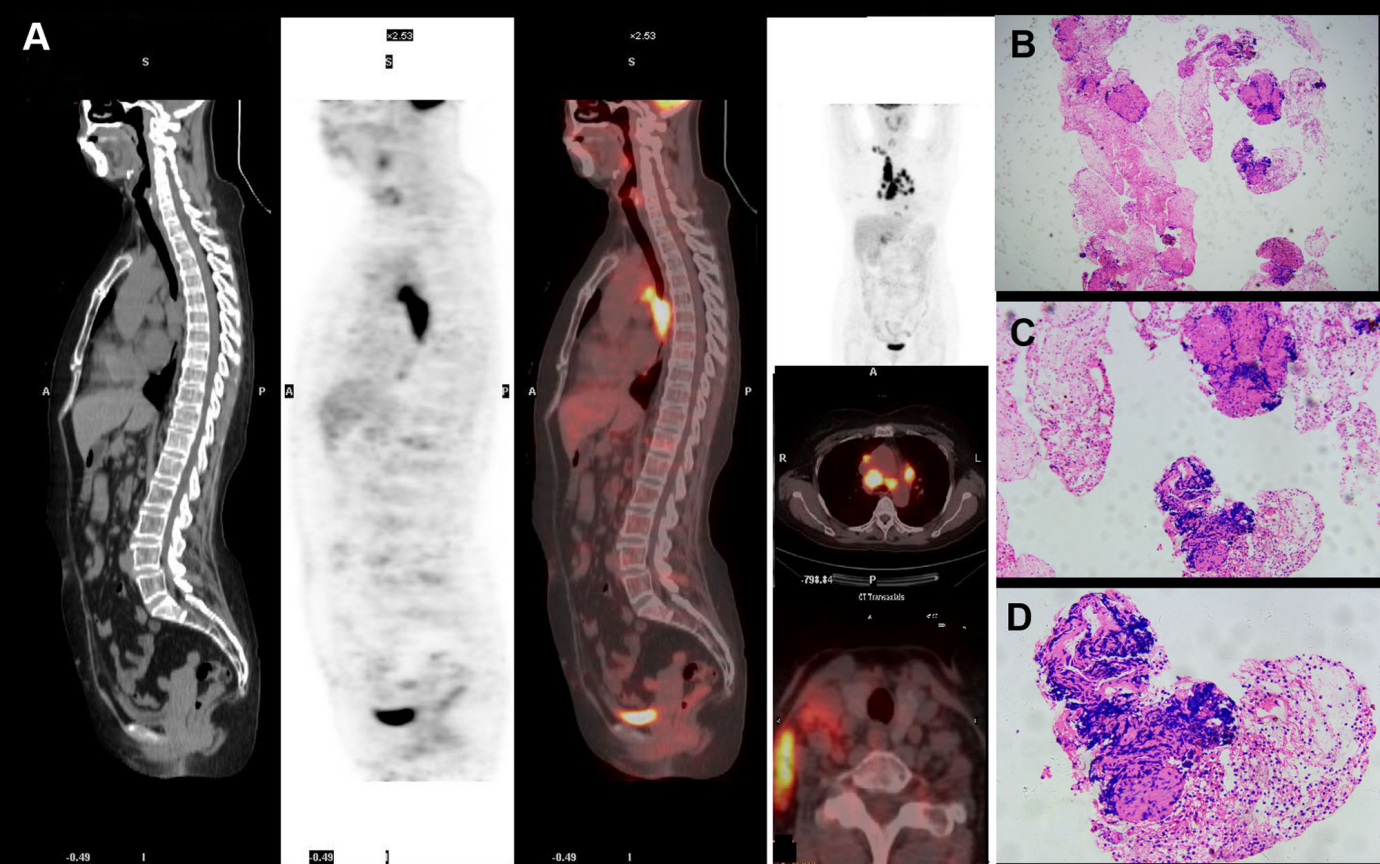
Imaging findings and biopsy of lymph nodes.PET-CT revealed multiple scattered enlarged lymph nodes with increased ¹^8^F-FDG metabolic activity in the right cervical, supraclavicular, bilateral hilar, and mesenteric regions **(A)**. Biopsy of a right cervical lymph node demonstrated focal epithelioid granulomas with admixed multinucleated giant cells, without caseous necrosis **(B)** ×100; **(C)** ×200; **(D)** ×400).

**Figure 4:**
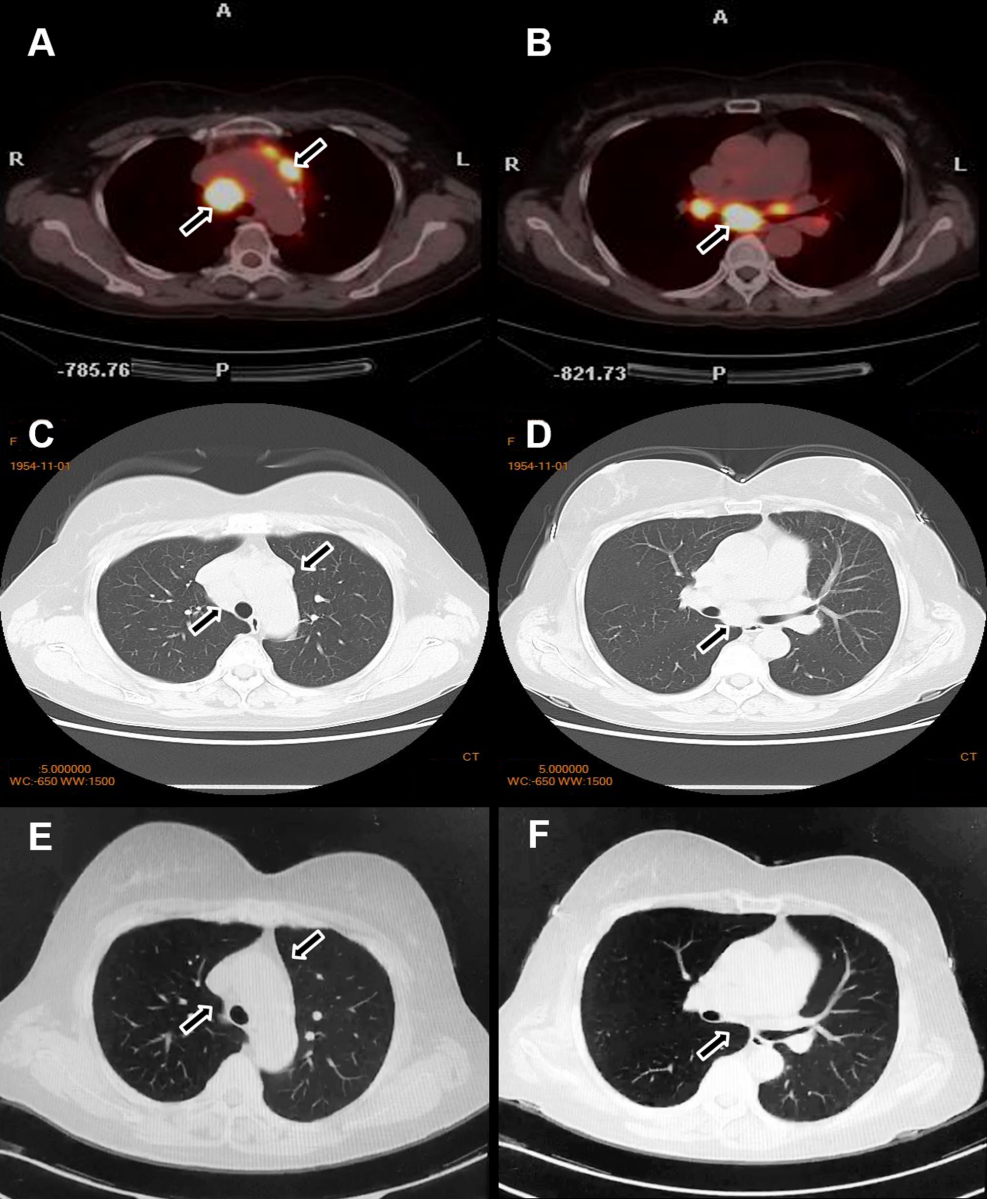
Changes in the lymph nodes before and after treatment. Before treatment, PET-CT **(A, B)** and chest CT **(C, D)** revealed enlarged lymph nodes in the bilateral supraclavicular and hilar regions. After treatment, a marked reduction in the size of the lymph nodes was observed **(E, F)**.

Chest CT revealed lymphadenopathy in the mediastinal and hilar regions. Subsequent PET-CT demonstrated multiple enlarged lymph nodes with increased ¹^8^F-fluorodeoxyglucose (FDG) metabolic activity in the right cervical, supraclavicular, bilateral hilar, and mesenteric regions (**Figure 3A**, and **Figures 4A–D**).

We obtained a 0.5 cm specimen through surgical excision from the lumbosacral region and right lower limb, which was then subjected to hematoxylin-eosin (HE) staining. Histologic examination of the former revealed hyperkeratosis, parakeratosis, loss of the granular layer, acanthosis, dilated tortuous blood vessels, and neutrophilic infiltration within the stratum corneum (Munro microabscesses), confirming the diagnosis of psoriasis (**Figures 1B-D**). Histopathological analysis of the latter demonstrated well-circumscribed epithelioid granulomas with multinucleated giant cells and sparse lymphocytic infiltration in subcutaneous tissue, without caseous necrosis. Acid-fast staining for Mycobacterium tuberculosis was negative (**Figures 2D-F**). Furthermore, to evaluate the nature of the lymphadenopathy, a fine-needle aspiration biopsy was performed on the enlarged right cervical lymph node, followed by HE staining, which revealed histological features consistent with those of the skin lesion. (**Figures 3B-D**). These findings were consistent with sarcoidosis. The final diagnosis confirmed psoriasis coexisting with sarcoidosis.

Although glucocorticoids are a first-line treatment for sarcoidosis, they are generally avoided in psoriasis due to the risk of transforming plaque psoriasis into more severe forms, such as erythrodermic or pustular psoriasis—particularly in cases of irregular medication adherence. Therefore, based on safety considerations, we opted to treat this patient with MTX, which effectively addresses both conditions. The patient was subsequently started on oral MTX 10 mg weekly. After three months of therapy, the cutaneous lesions of both psoriasis and sarcoidosis resolved completely. Repeat chest CT demonstrated significant regression of the enlarged pulmonary lymph nodes (**Figures 4E, F**). The patient was subsequently maintained on a low-dose MTX regimen (5mg/week). To date, the patient has remained clinically stable with no notable abnormalities.

## Discussion

Shared underlying immune dysregulation may play a pivotal role in the pathogenesis of psoriasis comorbidities (7). Sarcoidosis is a multisystem granulomatous disorder characterized by nonspecific clinical manifestations. Skin sarcoidosis has varied appearances, most commonly presenting as small bumps or broad, raised lesions—typically red-brown or purple—which may cause lasting discoloration or scarring (8). This case presented with characteristic annular nodules on both lower limbs—a rare manifestation in previous case reports that may mislead clinical diagnosis.

The co-occurrence of sarcoidosis and psoriasis may not be coincidental, potentially reflecting shared underlying immune pathways that drive both conditions (9). Current clinical studies have indicated a severity-dependent association between psoriasis and sarcoidosis, the relative strength of which may stem from overlapping immunopathological mechanisms and shared genetic susceptibility (10, 11). Additionally, case reports have highlighted that when patients present with psoriasiform skin lesions refractory to treatment, cutaneous sarcoidosis should be considered in the differential diagnosis (12). Notably, cases of sarcoidosis in patients with autoimmune diseases who were receiving anti-TNF-α agents have been reported (13). Here, our patient had no history of biologic medication application, so the occurrence of sarcoidosis was not associated with biologics in this patient.

The Th1/Th17-mediated immune response plays a pathogenetic role in psoriasis and sarcoidosis, potentially serving as a unifying mechanism in these diseases. In psoriasis pathogenesis, Th17-derived IL-17 stimulates keratinocyte hyperproliferation and aberrant differentiation. This cytokine further induces keratinocytes to secrete multiple chemokines and proinflammatory cytokines (11), which recruit neutrophils and other inflammatory cells to cutaneous sites. Such cellular infiltrates amplify local inflammation, ultimately driving the formation of characteristic psoriatic plaques (14). In sarcoidosis, Th17 cells may play a potentially critical role throughout all phases of granuloma evolution, encompassing granuloma formation, maintenance, and resolution (15, 16). The precise role and underlying mechanisms of Th17 cells in the co-occurrence of psoriasis and sarcoidosis remain incompletely characterized. Further investigations are warranted to delineate their pathobiological interrelationships.

## Conclusion

In summary, our case illustrates the coexistence of sarcoidosis in a patient with psoriasis. This suggests that shared underlying pathogenic mechanisms may explain the coexistence of psoriasis with these distinct conditions. We present the rare case to highlight their common pathophysiological links, aiming to improve the management of patients with psoriasis who develop sarcoidosis.

## Data Availability

The original contributions presented in the study are included in the article/supplementary material. Further inquiries can be directed to the corresponding authors.
